# Genomic evidence for a hybrid origin of the yeast opportunistic pathogen *Candida albicans*

**DOI:** 10.1186/s12915-020-00776-6

**Published:** 2020-05-06

**Authors:** Verónica Mixão, Toni Gabaldón

**Affiliations:** 1 Centre for Genomic Regulation, The Barcelona Institute of Science and Technology, Dr. Aiguader 88, 08003 Barcelona, Spain; 2 Life Sciences Department, Barcelona Supercomputing Center (BSC), Jordi Girona, 29, 08034 Barcelona, Spain; 3 Institute for Research in Biomedicine (IRB), The Barcelona Institute of Science and Technology, Barcelona, Spain; 4 Universitat Pompeu Fabra (UPF), Barcelona, Spain; 5 ICREA, Pg. Lluis Companys 23, 08010 Barcelona, Spain

**Keywords:** *Candida albicans*, Yeast, Pathogen, Hybrid, Genome

## Abstract

**Background:**

Opportunistic yeast pathogens of the genus *Candida* are an important medical problem. *Candida albicans*, the most prevalent *Candida* species, is a natural commensal of humans that can adopt a pathogenic behavior. This species is highly heterozygous and cannot undergo meiosis, adopting instead a parasexual cycle that increases genetic variability and potentially leads to advantages under stress conditions. However, the origin of *C. albicans* heterozygosity is unknown, and we hypothesize that it could result from ancestral hybridization. We tested this idea by analyzing available genomes of *C. albicans* isolates and comparing them to those of hybrid and non-hybrid strains of other *Candida* species.

**Results:**

Our results show compelling evidence that *C. albicans* is an evolved hybrid. The genomic patterns observed in *C. albicans* are similar to those of other hybrids such as *Candida orthopsilosis* MCO456 and *Candida inconspicua*, suggesting that it also descends from a hybrid of two divergent lineages. Our analysis indicates that most of the divergence between haplotypes in *C. albicans* heterozygous blocks was already present in a putative heterozygous ancestor, with an estimated 2.8% divergence between homeologous chromosomes. The levels and patterns of ancestral heterozygosity found cannot be fully explained under the paradigm of vertical evolution and are not consistent with continuous gene flux arising from lineage-specific events of admixture.

**Conclusions:**

Although the inferred level of sequence divergence between the putative parental lineages (2.8%) is not clearly beyond current species boundaries in Saccharomycotina, we show here that all analyzed *C. albicans* strains derive from a single hybrid ancestor and diverged by extensive loss of heterozygosity. This finding has important implications for our understanding of *C. albicans* evolution, including the loss of the sexual cycle, the origin of the association with humans, and the evolution of virulence traits.

## Background

Hybrids are chimeric organisms that originate from the cross between two diverged lineages (whether from the same or distinct species). At the time of hybridization, the divergence at the sequence level between the pairs of homeologous chromosomes is similar to the divergence between the parental genomes. Consequently, hybrid genomes have high levels of heterozygosity, which can subsequently be eroded through recombination-mediated conversion of homeologous sequences, resulting in loss of heterozygosity (LOH, [1–4]). Hybridization may produce organisms with unique phenotypic features, which may contribute to the successful adaptation to new niches, being often associated with speciation [5–8]. Many hybrids have been described in animals and plants. Examples go from butterflies to birds, nematodes, or even sunflowers [9–12]. In fungi, advances in next-generation sequencing have recently allowed the identification of many hybrid lineages as well [13, 14], of which some have importance for biotechnology and food or beverage industries [15–17]. Hybrids with medical relevance have also been described, particularly in *Cryptococcus* and *Candida* clades [1–3, 18–20], with earlier studies suggesting that hybridization might be related to the emergence of virulence traits in some of these species [1–4].

*Candida* species are the most common causative agents of hospital-acquired fungal infections [21–25], accounting for 72.8 million opportunistic infections per year, with an overall mortality rate of 33.9% [21, 26]. *Candida albicans* is a commensal organism that can form part of the microbiota of healthy individuals [27]. Under certain circumstances, such as a weakening of the host immune system, *C. albicans* can shift from commensal to pathogenic behavior [21]. This species is the causative agent in more than 50% of the candidaemia cases worldwide [21]. Although *C. albicans* cannot undergo a normal sexual cycle involving meiosis, it is known to be able to follow a so-called parasexual cycle [28, 29]. This consists of the mating of two diploid cells, forming a tetraploid cell that subsequently returns to a diploid state by concerted chromosomal loss. Restoration of the diploid state is not always properly achieved, leading to aneuploidies [29, 30]. Thus, this system constitutes a source of genetic variability, which has been proposed to be advantageous under stress conditions [29]. Although the ability to undergo a sexual or parasexual cycle has not been thoroughly investigated in non-albicans *Candida* species, accumulating evidence suggests that some forms of mating and genomic recombination might be common even in species traditionally considered as asexual [31].

Recently, the genomic diversity of *C. albicans* strains belonging to different MLST-based clades and isolated from globally distributed locations was investigated [32–36]. These studies have shown that the *C. albicans* genome shows signs of recombination, with genomic material exchanged between different strains [34, 35]. Furthermore, they have reported that the fraction of the genome covered by heterozygous regions can vary between 48 and 89%, depending on the strain, and that these heterozygous tracts are separated by regions of LOH [33–36]. Moreover, it has been shown that the accumulation of mutations and the exchange of genomic material between strains are the main forces shaping *C. albicans* genome [35].

However, an intriguing and still unaddressed question is: what is the initial source of the high heterozygosity levels present in *C. albicans* genome? Can the accumulation of mutations over long periods of time and the presence of inter-strain recombination explain the levels of heterozygosity observed in highly heterozygous regions of *C. albicans* strains? We noted that the genomic patterns observed in *C. albicans* are reminiscent of recently analyzed yeast hybrid species, such as *Candida inconspicua*, *Candida orthopsilosis*, and *Candida metapsilosis* [2, 3, 20, 34, 37]. Hence, a possible scenario for the observed genomic patterns in *C. albicans* is that the divergence observed in highly heterozygous regions is not exclusively the consequence of continuous accumulation of mutations within the lineage, but also, to a large degree, a footprint of an ancient hybridization event between two diverged lineages. We here put these alternative hypotheses at test by comparing *C. albicans* genomic patterns with the ones observed in *C. inconspicua*, *C. orthopsilosis*, and *C. metapsilosis* hybrid strains, as well as, non-hybrid strains from these and other species.

## Results

### *K*-mer profiles of *C. albicans* sequencing libraries reveal a heterogeneous content similar to that of hybrid genomes

**Fig. 1:**
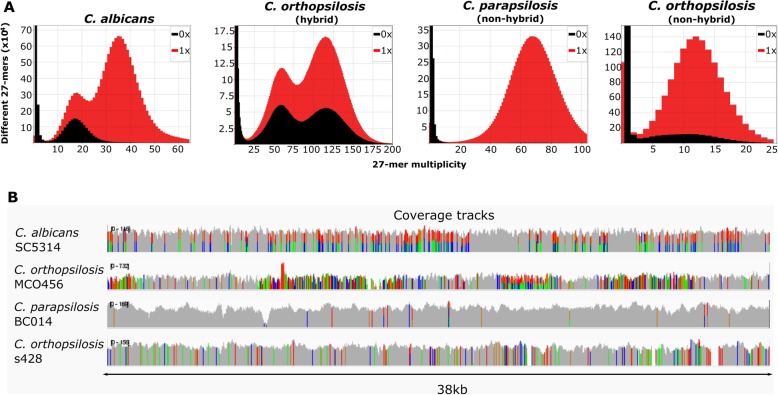
Comparison of the genomic patterns observed in *C. albicans* and hybrid and non-hybrid species. **a** The 27-mer frequency for SC5314 (*C. albicans*), MCO456 (*C. orthopsilosis* hybrid), BC014 (*C. parapsilosis* non-hybrid), and s428 (*C. orthopsilosis*, non-hybrid parental lineage A), and their respective presence (red) or absence (black) in the respective reference genome (plots were obtained with KAT [38]). **b** Coverage tracks for illustrative genomic regions of the abovementioned genomic sequencing libraries, when aligned to the respective genomes. Colors indicate polymorphic positions. Positions with more than one color correspond to heterozygous variants. Visualizations were performed with IGV [39]

To assess heterozygosity levels in *C. albicans* genomes, we analyzed 27-mer frequencies of raw sequencing data of Illumina paired-end libraries from different *C. albicans* strains (see the “Methods” section). All analyzed sequencing libraries produced similar 27-mer profiles, showing two peaks of depth of coverage, one with half coverage of the other, which corresponded to heterozygous and homozygous regions, respectively (Fig. 1a and Additional file 1: Fig. S1). Of note, for all strains, including the reference, approximately half of the 27-mers of the first peak were not represented in the reference assembly (Fig. 1a and Additional file 1: Fig. S1) and therefore could correspond to heterozygous regions where only one of the haplotypes was represented in the reference assembly. This bimodal pattern was also produced by sequencing libraries from hybrid strains of *C. orthopsilosis* and *C. metapsilosis* mapped to their respective reference genomes (Fig. 1a and Additional file 1: Fig. S1) and was previously reported for *C. inconspicua* hybrids [3]. However, this pattern was not observable in sequencing libraries from non-hybrid strains of *Candida dubliniensis*, *Candida tropicalis*, *C. orthopsilosis*, and *Candida parapsilosis* (Fig. 1a and Additional file 1: Fig. S1). As shown in Additional file 1: Fig. S1, hybrid strains with lower levels of heterozygosity (i.e., strains from *C. orthopsilosis* clade 1) had a higher homozygous peak as compared to hybrid strains with higher levels of heterozygosity (i.e., *C. orthopsilosis* clade 4). Altogether, these results show that the relative frequency of the two peaks is representative of the level of heterozygosity in hybrid genomes and that the patterns observed in genomes from *C. albicans* strains are similar to those of hybrid strains of *C. orthopsilosis* clade 1 (e.g., strain MCO456), which underwent extensive levels of LOH after hybridization [20, 37].

### Heterozygosity patterns in *C. albicans* are comparable to those of hybrid lineages

We next assessed heterozygosity levels by aligning genomic reads of *C. albicans* strains, including putative wild strains isolated from oak trees [36], on the reference for haplotypes A and B, independently (see the “Methods” section). Similar results were obtained for the two haplotypes, and therefore, we only report the results for haplotype A. This analysis revealed a genome-wide average heterozygosity of 6.70 heterozygous variants per kilobase (kb), which depending on the clade varied from 4.59 to 8.62 variants/kb (Additional file 2: Table S1). Once again, these values are comparable to what was observed for *C. orthopsilosis* MCO456 (clade 1) strain, where we found 7.16 heterozygous variants/kb (Additional file 3: Table S2). Of note, *C. albicans* strains isolated from oak trees were highly heterozygous, as previously reported [36], but their values were in the range of what we observed for clinical isolates.

Furthermore, as previously described [32, 34–36], the heterozygous variants in *C. albicans* were not homogeneously distributed across the genome. Rather, these variants were concentrated in heterozygous regions separated by what appeared to be blocks of LOH (Fig. 1b and Additional file 4: Fig. S2). Moreover, we could identify some regions which were highly heterozygous in some strains whereas in others corresponded to LOH regions that tended to alternative haplotypes (Additional file 4: Fig. S2). These patterns were reminiscent of the ones observed in *Candida* hybrid species (Fig. 1b and Additional file 4: Fig. S2) [2, 3, 20, 37].

The comparison of heterozygosity patterns between different species is often hampered by the use of different methodologies and criteria to define heterozygous and homozygous regions in different studies. For instance, while studies performed so far on *C. albicans* distinguished heterozygous from LOH regions based on SNP density within windows of 5 kb [32, 33, 35], 10 kb [34], or even 100 kb length [36], studies performed on *Candida* spp. hybrids defined these blocks based on the distance between heterozygous positions [2, 3, 20]. This last approach makes the boundaries between heterozygous and LOH blocks more flexible and precise and avoids averaging levels of heterozygosity when a window spans both homozygous and heterozygous regions. We therefore decided to use the methodological framework previously applied and validated in the study of hybrid species (see the “Methods” section) to analyze genome-wide heterozygosity patterns and define LOH blocks in *C. albicans* and compare them to patterns in established *Candida* hybrids.

On average, in *C. albicans* strains, we could define 7059 heterozygous blocks and 16,492 LOH blocks, representing 11.18% and 85.74% of the genome, respectively. This is again notably similar to *C. orthopsilosis* hybrid clade 1, where 84.85% of the genome underwent LOH (Additional file 3: Table S2 and Additional file 5: Tables S3). Although these heterozygous blocks in *C. albicans* comprised most of the heterozygous SNPs (on average 59.35%), 12.79% of the heterozygous variants were placed within LOH blocks, and the remaining 27.86% in undefined regions (see the “Methods” section). The number of heterozygous variants outside heterozygous blocks was comparatively much higher than those found in *C. orthopsilosis* or *C. metapsilosis* hybrids (where it ranged from 2.38 to 5.81%, depending on the strain, Additional file 3: Table S2), but notably closer to that found in the recently identified *C. inconspicua* hybrids (up to 23.23%, [3]). The higher number of heterozygous variants within LOH blocks would suggest that *C. albicans* and *C. inconspicua* LOH blocks have been accumulating mutations for a longer time, as compared to *C. orthopsilosis* or *C. metapsilosis*. In addition, it is worth noting that the union of the heterozygous blocks of the 61 *C. albicans* strains analyzed in this work corresponded to more than half (53.17%, Additional file 6: Table S4) of the *C. albicans* genome, and this value is expected to increase with a larger sample size. Although from these analyses, we cannot completely exclude the possibility that the accumulation of mutations followed by recombination between different strains is responsible for the heterozygosity in *C. albicans* [34, 35], their strong similarity with what is observed for *C. inconspicua*, *C. orthopsilosis*, and *C. metapsilosis* hybrid strains and the high level of heterozygosity of *C. albicans* genome strongly suggest a scenario where hybridization between two diverged lineages was followed by extensive LOH.

### The majority of heterozygous variants in *C. albicans* predate the diversification of known clades

**Fig. 2:**
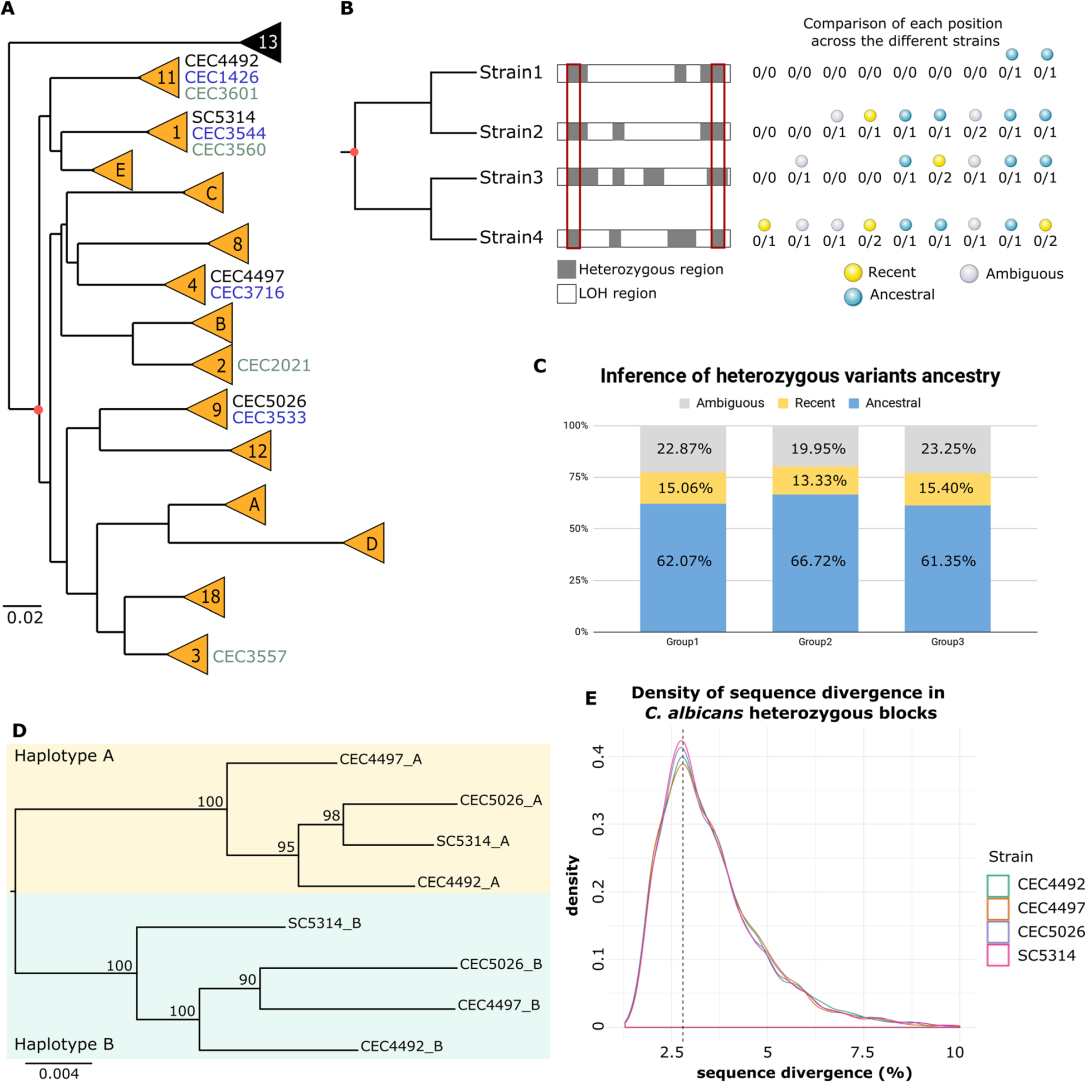
Analysis of *C. albicans* heterozygosity patterns. **a** Schematic phylogenetic tree adapted from [40] indicating the different *C. albicans* clades in orange. The potential common ancestor is marked with a red dot. Strains used for the comparison of heterozygous positions across different clades are indicated, with strains of the same group of analysis being written with the same color (black—group 1, blue—group 2, and gray—group 3). **b** Schematic representation of the methodology for the comparison of heterozygous SNPs in heterozygous blocks (gray). The intersection of the heterozygous blocks is represented by the red rectangle (although not shown, the same approach was used for the analysis of LOH blocks). Examples of possible combinations of genotypes across the four strains are given (“0”—allele similar to the reference, “1”—allele different from the reference, “2”—allele different from the reference and from “1”). The most parsimonious path for the SNPs observed in each position was reconstructed. The decision taken for each position in a given strain is represented by yellow (recent), blue (ancestral), or gray (ambiguous) spheres. **c** Plot of the average proportion of ambiguously (gray) and unambiguously assigned positions for each group of strains. For unambiguously assigned positions, the proportion of recent and ancestral positions is shown in yellow and blue, respectively. **d** Maximum likelihood phylogeny of the aligned reconstructed haplotypes A and B for the intersection of heterozygous blocks > 100 bp of group 1. **e** Sequence divergence distribution in heterozygous blocks of *C. albicans* SC5314, CEC4492, CEC4497, and CEC5026

The level of conservation of heterozygosity patterns across *C. albicans* strains of different clades can be used to assess the possibility of an ancestral hybridization event. Indeed, if a hybridization event between two divergent lineages, rather than the vertical accumulation of variants, was responsible for a sizable fraction of the heterozygosity levels observed across *C. albicans* genomes, then we would expect that a significant amount of heterozygous SNPs within heterozygous blocks would be shared by strains from deeply divergent clades, as their origin would have predated the diversification of the different *C. albicans* clades. To test this, we selected three non-overlapping sets of four strains (considered as replicates, see the “Methods” section) so that each set contains a representative strain of each of four deeply divergent clades (Fig. 2a), according to the recent strain phylogeny described by [34]. For each group, we compared the heterozygous positions in heterozygous and LOH regions shared by the different clades (Fig. 2b, see the “Methods” section). We inferred that a heterozygous position in a given strain was ancestral if the most parsimonious reconstructed scenario (i.e., the one involving the lowest number of mutations) pointed to the same heterozygous genotype (i.e., the combination of the same two alleles) in the common ancestor of the four clades. The results, considering positions that could be unambiguously inferred, were consistent between the different groups (Fig. 2c, Additional file 7: Table S5) and showed that on average, between 79.93 and 83.34% of the heterozygous positions within heterozygous blocks were ancestral (i.e., were present before the divergence of the clades), as compared to 15.28 to 20.10% of heterozygous positions within LOH blocks. Interestingly, the density of ancestral SNPs supporting a hybridization scenario presented a normal distribution with a peak at 20 SNPs/kb in all strains (Additional file 8: Fig. S3). The high level of common SNPs in heterozygous regions is shared between different clades even when considering coding and non-coding regions separately (Additional file 7: Table S5). These results strongly suggest that most of the heterozygous variants in heterozygous blocks were already present in a putative *C. albicans* ancestor, whereas most of the variants in LOH blocks appeared later, by independent accumulation in the different lineages.

In agreement with these results, a maximum likelihood phylogeny of reconstructed haplotypes in heterozygous regions for the same groups of four strains (where A and B refer to SC5314 described haplotypes, see the “Methods” section) indicated that the phylogenetic distance between the two haplotypes is higher than the distance between the different strains (Fig. 2d and Additional file 9: Fig. S4). A similar approach was used in the past to confirm a hybrid origin of *C. orthopsilosis* [20]. Of note, the specific topological arrangement between strains is different for each of the two haplotypes, which supports the existence of recombination between haplotypes.

Based on the number of variants per kilobase in heterozygous blocks, we estimated that the homeologous chromosomes are currently approximately 3.5% divergent at the nucleotide level (Fig. 2e and Additional file 5: Table S3). This estimation was consistent across the sixty-one different strains (ranging from 3.44 to 3.59%, Additional file 5: Table S3) and therefore unrelated to their overall level of heterozygosity. These analyses strongly suggest that most of the divergence between haplotypes in *C. albicans* heterozygous blocks was already present in a putative, highly heterozygous ancestor, with an estimated 2.8% divergence between the homeologous chromosomes (assuming ~ 80% of the current variants in heterozygous blocks were heterozygous in the ancestor, in line with our estimations above). We consider that vertical accumulation of such level of heterozygosity across the entire genome is not plausible, as it would imply extremely long periods of mutation accumulation in the absence of any inter-strain recombination or LOH, events which have been shown to be common in this species [34]. Therefore, we consider that the most likely scenario to explain such a pattern is a hybridization event between two divergent lineages thereby forming a highly heterozygous ancestor.

### *Candida africana* is not a putative parental of the hybrid ancestor of *C. albicans*

*C. africana* was proposed to be ranked as a species in 2001 [41]. However, although it presents particular phenotypes, the genetic similarity with *C. albicans* makes this controversial, and *C. africana* is often considered another *C. albicans* clade (clade 13, [42]). In a recent population genomics study, Ropars et al. showed that contrary to *C. albicans*, *C. africana* is highly homozygous, and hypothesized that massive LOH might have occurred in this clade [34].

Given our results indicating that *C. albicans* originates from a hybridization event, we wanted to investigate the possibility that *C. africana* lineage could correspond to one of the parentals involved in the hybridization. In the previously described *C. orthopsilosis* hybrids, for which one of the parental lineages is known, both MATa and MATalpha alleles in the hybrids exhibit a level of sequence divergence between the two parentals that is similar to that of the remaining nuclear genome [37, 43]. Additionally, for each hybrid clade, when the two mating loci are present, only one of them is similar to the parental strain, with the other having high divergence indicating it descends from the other parental. Therefore, if *C. africana* was indeed one of *C. albicans* parentals, we would expect only one of the alleles to be inherited by the ancestral hybrid. In this case, we would expect only one of the two mating type loci in *C. albicans* clades to be similar to *C. africana*. Contrary to this expectation, our analysis reveals that both MATa and MATalpha of *C. africana* are highly similar (0.21% and 0.25% divergence, respectively) to those of *C. albicans*. This suggests that both *C. africana* and *C. albicans* share the same MAT locus alleles, and therefore, *C. africana* is not a parental species but rather another descendant from the same hybrid ancestor.

To confirm this, we selected a sample of *C. africana* strains (see the “Methods” section) and analyzed the respective genomic patterns. As expected, given the high levels of LOH previously described for this lineage [34], *C. africana* presented low levels of heterozygosity, with an average of 2.68 heterozygous variants/kb, which were still high when compared to non-hybrid strains (Additional file 10: Table S6). Therefore, we decided to define heterozygous regions in *C. africana* strains. In contrast to *C. albicans*, only 3.8% of the genomes, on average, corresponded to heterozygous blocks. These blocks have a current haplotype divergence of 3.7%, which is close to the 3.5% mentioned above for *C. albicans* (Additional file 10: Table S6).

Furthermore, if *C. africana* was one of *C. albicans* parents, we would expect the homozygous regions of a given chromosome to tend to correspond always to the same *C. albicans* haplotype. The available phased genome of *C. albicans* is based on the heterozygous strain SC5314 [44], which already underwent LOH. Thus, only the regions of the phased reference genome corresponding to heterozygous regions of this strain can be used to assess distinct haplotypes in the inferred ancestral hybrid. From these regions, we selected heterozygous positions that were considered ancestral in the abovementioned analyses and compared them to homozygous regions in *C. africana* strains (see the “Methods” section for details). Our results indicate that similar proportions of homozygous positions in *C. africana* could be mapped to each of the two haplotypes (55% A and 45% B, where A and B refer to SC5314 haplotypes, Additional file 11: Table S7). This suggests that each *C. africana* chromosome is a mosaic of the two SC5314 haplotypes. Although, due to recombination between ancestral haplotypes (see above), SC5314 haplotypes might be chimeric in relation to the ancestral parental genomes, this result, together with the existence of heterozygous blocks in *C. africana* genome, provides support for a scenario considering massive LOH from a common highly heterozygous ancestor. A shared ancestral hybridization scenario is reinforced by the fact that for the majority (99%) of the ancestral *C. albicans* heterozygous positions, one of the alleles was represented in *C. africana.*

### Continuous gene flux from divergent lineages cannot explain consistent patterns found across strains

**Fig. 3:**
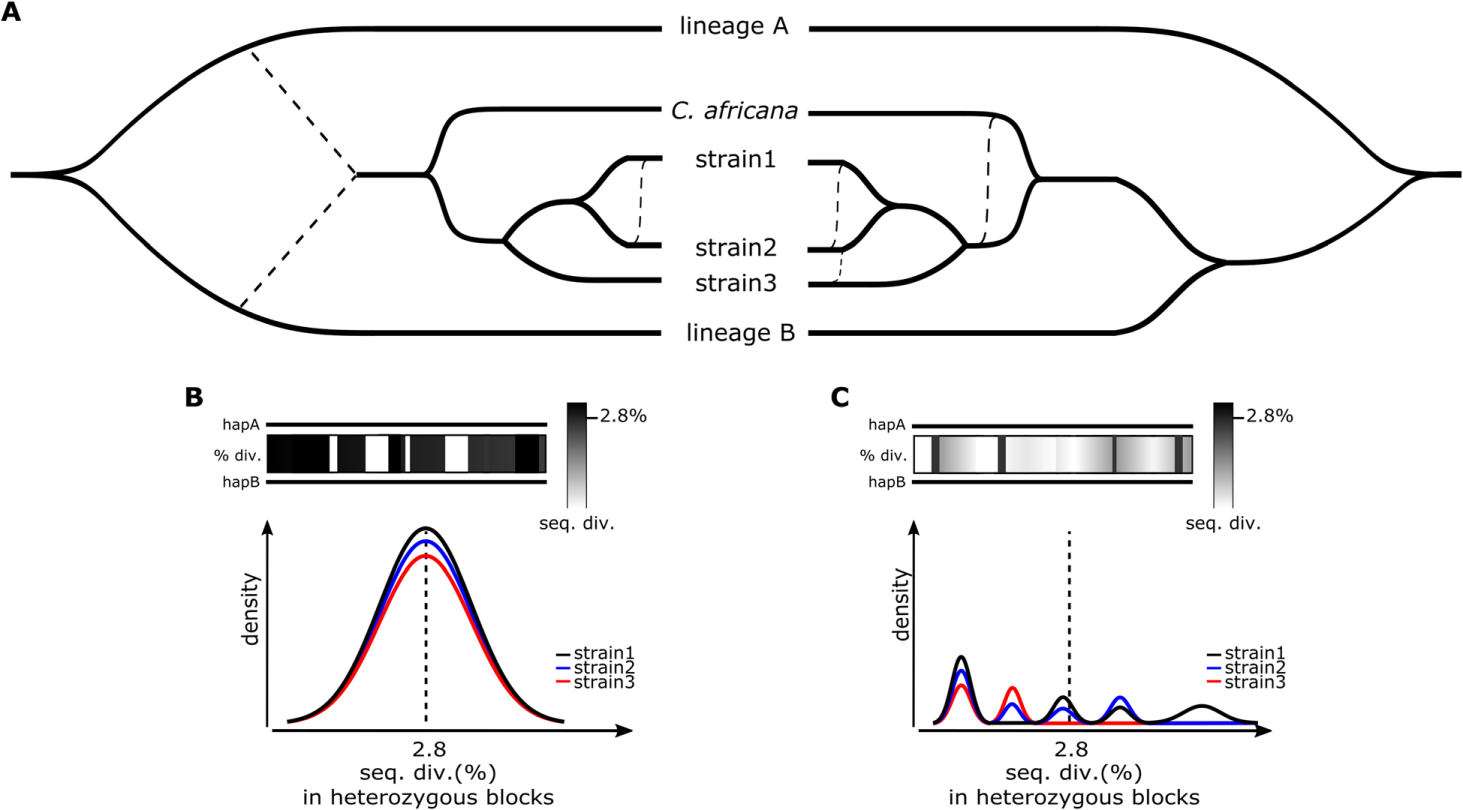
Schematic representation of plausible scenarios for the origin of *C. albicans* heterozygosity. **a** The scenario proposed by this work is presented on the left, showing an ancestral hybridization event between two diverged lineages as the main source of *C. albicans* heterozygosity, followed by LOH, particularly extensive in *C. africana*, and more recent exchange of genomic material between *C. albicans* strains (dashed lines). The alternative scenario is presented on the right, showing inter-strain recombination (dashed lines) as the only explanation for the heterozygous patterns observed in *C. albicans*. **b** Scheme of the expected sequence divergence patterns between the two haplotypes if a single hybridization event was in the origin of the heterozygosity in *C. albicans*. After a hybridization event, the heterozygous blocks are expected to have similar sequence divergence, which is then reflected in a normal distribution. This divergence is expected to be similar in all strains originated from the same event. **c** Scheme of the expected sequence divergence patterns if inter-strain recombination was the only source of variability in *C. albicans*. In this scenario, the sequence divergence is time and strain dependent, and therefore, different patterns are expected between different blocks and between different strains

Taken together, our results show compelling evidence for a highly heterozygous genome in the ancestor of currently sampled *C. albicans* and *C. africana* clades. The presence of highly divergent haplotypes and its consistency over the entire *C. albicans* genome can be best explained by an ancestral hybridization event between two distinct lineages and subsequent evolution through LOH. The alternative scenario of continuous introgression between divergent lineages could as well explain the presence of heterozygous regions in *C. albicans* strains but could not explain the observed similarity of heterozygosity patterns across strains (Fig. 3). Indeed, in a single hybridization scenario followed by divergence, extant heterozygous blocks in divergent strains are expected to present similar levels of sequence divergence, to share the same ancestral heterozygous SNPs, and to show some degree of overlap in their patterns of heterozygosity, as we have described. Moreover, we expect similar levels of heterozygosity and LOH between the strains from the same hybridization event [37]. Such consistent features of heterozygous blocks across divergent strains are difficult to explain by the exclusive accumulation of lineage-specific and independent events of admixture, because sequence divergence is expected to be time dependent, and therefore, different events would leave different tracks (Fig. 3c). Importantly, an ancestral hybridization scenario not only readily explains the heterozygosity patterns found in extant strains, but also could provide an explanation for the origin of other peculiar characteristics of *C. albicans* such as the absence of a standard sexual cycle, or its ubiquitous diploid nature, as discussed further below.

## Discussion

Advances in next-generation sequencing have recently allowed the identification of many hybrid fungi with clinical relevance [2, 3, 19, 20, 37]. Hybridization between diverged lineages is known to have an important role in the adaptation to new environments, or even in the emergence of new pathogens, as it is hypothesized to be the case of *C. metapsilosis* and *C. inconspicua* [1–3]. The hybrid nature of these strains was discovered by noting the presence of highly heterozygous genomes with a large divergence between alternative haplotypes and showing characteristic non-homogeneous distributions of heterozygous variants, resulting in highly heterozygous blocks separated by regions of low heterozygosity, likely resulting from LOH events. In most such cases, these hybrid strains are diploid and seem to be unable to undergo a normal sexual cycle [20].

*C. albicans*, the most important yeast pathogen for human health [45], was previously shown to present genomic regions with high heterozygosity separated by what appeared to be blocks of LOH [34]. Parasex and admixture were taken as the possible source for the observed levels of heterozygosity [34, 35]. However, as the description of these patterns was reminiscent of that observed in hybrid lineages [2, 3, 37], we hypothesized that hybridization could have been the initial source of genomic variability in *C. albicans*. Our results show compelling evidence that *C. albicans* descend from a hybrid between two divergent lineages. The genomic patterns observed in this pathogen are similar to those of other hybrids, especially *C. orthopsilosis* MCO456 and *C. inconspicua* [3, 20, 37]. This scenario clearly points to a highly heterozygous ancestor predating the divergence of currently known clades. The reconstructed most recent common ancestor of sequenced *C. albicans* strains would present most (53.17%) of its genome within heterozygous blocks. Current heterozygous regions present a 3.5% divergence between the two haplotypes, and we here infer that around 80% of such variants are ancestral, suggesting that the putative *C. albicans* ancestor had, at least, roughly 2.8% sequence divergence at the nucleotide level, and that this hybridization event is relatively older when compared to other hybrids, such as *C. orthopsilosis* or *C. metapsilosi*s [2, 37]. From our analyses, we conclude that the existence of such level of divergence between two haplotypes in heterozygous regions can be better explained by these two haplotypes being genetically isolated for a long time. A hybridization between two previously isolated lineages, followed by LOH and further accumulation of SNPs, would readily explain the observed patterns in *C. albicans*. Alternative scenarios accounting for the origin of heterozygous regions through independent introgression events would not explain the widespread presence of conserved heterozygous SNPs across strains from deeply divergent clades, nor the normal distribution observed for the levels of sequence divergence between haplotypes (Fig. 2e).

We want to stress that the scenario of the hybridization between divergent lineages is agnostic to the consideration of the parental lineages as different or the same species. What is relevant is the realization of genomic chimerism predating the divergence of *C. albicans* clades. The species concept in microbes is controversial. The estimated 2.8% ancestral divergence between the hybridizing lineages largely exceeds levels of divergence found between most distantly related strains of well-studied yeast species such as *Sacharomyces cerevisiae*, where 1.1% sequence divergence was found between the most distantly related strains [46], and is higher than the estimated divergence between different described fungal species such as 1.4% between *Verticillium dahliae* and *Verticillium longisporum* D1 parental [47, 48]. On the other hand, it can be considered low when compared to ~ 4.6% divergence between distant strains in *Saccharomyces paradoxus* [49]. Independently of the consideration of this putative ancestor as an inter- or intra-species hybrid, our results indicate that the ancestral hybrid did not backcross significantly with any of the parental lineages but rather further evolved in a mostly clonal manner.

Inability to undergo meiosis and to complete a sexual cycle is a common feature of hybrids [50, 51], including intra-species ones [52, 53]. Considering this, a hybridization scenario for the origin of *C. albicans* lineages could provide a plausible explanation for the origin of the inability of *C. albicans* to sporulate or undergo a standard sexual cycle. In this particular case, it could be hypothesized that the improper chromosome pairing after hybridization and consequent impossibility of completing meiosis contributed to the development of a parasexual cycle, an essential mechanism for *C. albicans* genomic plasticity. Alternatively, parasex might be a more ancient trait in the clade, predating the origin of the proposed hybridization. Supporting this is the finding that the closely related species *C. tropicalis* has also been shown to undergo a parasexual cycle under laboratory conditions [54]. If that is the case, hybridization between two lineages might have occurred through a sexual or parasexual cycle. Mating between different *Candida* species has been observed in the laboratory [55], although it is unclear how widespread is this ability among *Candida*. Determining the exact mechanism of hybridization is beyond the scope of our study. However, based on our observations, we favor scenarios, such as standard sexual mating, in which two haploid cells fuse to form a diploid hybrid. Indeed, the finding that heterozygous regions are widespread across the genome and present in all chromosomes is at odds with expectations from parasexual crossing. In parasex, two diploid cells fuse to form an unstable tetraploid that quickly returns to a diploid state through concerted chromosomal loss. This process would rarely yield a chromosomal set composed of a copy from each parent for all the chromosomes, whereas this is what is expected by fusion of haploid cells. Although *C. albicans* belongs to a clade of diploid yeasts, many diploid yeast species form haploid cells to undergo mating. Furthermore, a viable mating-competent haploid state has been demonstrated for *C. albicans* [56]. We believe that further research is needed to clarify whether asexuality is a result or a facilitator of hybridization in this and other hybrid cases.

Our results raise once more the question of the importance of hybridization for the emergence of yeast pathogens [1] and pose the intriguing question of whether *C. albicans* ability to colonize and infect humans was an emerging phenotype enabled by this hybridization event. Indeed, hybridization is an important evolutionary mechanism that generates highly heterozygous genomes. This heterozygosity is often a source of genomic plasticity that allows the emergence of new phenotypes or even adaptation to new niches. Many examples on different fungal species have showcased the relevance of hybridization for adaptation or diversification [4, 14, 57, 58]. From a clinical perspective, hybridization can be regarded as a source of new potentially pathogenic lineages [1]. Many hybrids are becoming important agents of human infection, as it is the case of *Candida* or *Cryptococcus* species [2, 3, 18–20, 37], with hybridization being also associated to a possible increase in virulence [4]. In a world where globalization and global warming are a reality promoting the expansion of certain species to locations where they have never been, the chances of new events of hybridization are possibly increasing. In this context, the impact of such events for public health should be regarded with some concern [59]. In the particular case of the *Candida* clade, multiple hybridization events leading to the emergence of pathogenic lineages have been described [2, 3, 37]. This shows that this clade has some propensity to hybridize and contribute to the emergence of pathogens. The reason why this happens is difficult to be addressed. More studies should be performed to clarify this question and uncover the mechanisms of success of these pathogenic hybrid lineages.

## Conclusion

This work assessed the origin of the high levels of heterozygosity in the important opportunistic pathogen *C. albicans*. We compared the genomic patterns of different strains and showed that *C. albicans* has a hybrid ancestor. This species is not the first hybrid lineage described in the *Candida* clade, but it is by far the most important one for the clinical setting. Why this clade has so many hybrid lineages is still unknown, but more studies should be performed trying to understand the particularities that make this clade so prone to hybridize. This finding raises once more the question about an apparent link between hybridization events and the emergence of pathogenic lineages. Therefore, future studies addressing the origins of pathogenicity should consider the contribution of non-vertical evolution to this event.

## Methods

### NGS data selection

*C. albicans* paired-end reads used in this work are a subset of the data made publicly available under the BioProjects PRJNA432884 and PRJEB27862 [34, 36]. Our sample was chosen based on different criteria. Specifically, we selected at least one strain from each SNP-based clade defined by Ropars et al., including *C. africana*, as this clade is highly homozygous [34] and could correspond to a putative parental lineage. For clusters with more than one site of collection, one strain from each site was taken. In addition, the *C. albicans* type strain and two other environmental isolates were retrieved from [36]. In the end, our sample consisted of a total of 61 *C. albicans* strains and eight *C. africana* (Additional file 2: Table S1).

In order to compare our results with other species, we retrieved raw reads of Illumina paired-end sequencing libraries from known hybrid and non-hybrid strains from diverse *Candida* species. As the representative of hybrid strains, we selected *C. orthopsilosis* MCO456 (BioProject PRJEB4430, SRA ERX295059); s425, s433, and s498 strains (BioProject PRJNA322245, SRA SRX1776098, SRX1776103, and SRX1776124); and *C. metapsilosis* CP367 (BioProject PRJEB1698, SRA ERX221928) [2, 20, 37]. As the representative of non-hybrid strains, we selected *C. orthopsilosis* s428 (BioProject PRJNA322245, SRA SRX1776102), three *C. parapsilosis* strains (BioProjects PRJEB1685 and PRJNA326748, SRA ERX221039, ERX221044, and SRX1875155), and *C. tropicalis* ATCC200956 (BioProject PRJNA194439, SRA SRR868710) [37, 60, 61].

### Library preparation and genome sequencing

As we considered it important to compare *C. albicans* with the closely related species *C. dubliniensis* and *C. tropicalis*, we decided to sequence two strains from our lab collections, namely 60-13 (*C. dubliniensis*) and CSPO (*C. tropicalis*). Genomic DNA extraction was performed using the MasterPure Yeast DNA Purification Kit (Epicentre, USA) following the manufacturer’s instructions. Briefly, cultures were grown in an orbital shaker overnight (200 rpm, 30 °C) in 15 ml of YPD medium. Cells were harvested using 4.5 ml of each culture by centrifugation at maximum speed for 2 min, and then, they were lysed at 65 °C for 15 min with 300 μl of yeast cell lysis solution (containing 1 μl of RNAse A). After being on ice for 5 min, 150 μl of MPC protein precipitation reagent was added into the samples, and they were centrifuged at 16,000*g* for 10 min to pellet the cellular debris. The supernatant was transferred to a new tube; DNA was precipitated using 100% cold ethanol and centrifuging the samples at 16,000*g*, 30 min, 4 °C. The pellet was washed twice with 70% cold ethanol, and once the pellet was dried, the sample was resuspended in 100 μl of TE. All gDNA samples were cleaned to remove the remaining RNA using the Genomic DNA Clean & Concentrator kit (Epicentre) according to the manufacturer’s instructions. Total DNA integrity and quantity of the samples were assessed by means of agarose gel, NanoDrop 1000 Spectrophotometer (Thermo Fisher Scientific, USA), and Qubit dsDNA BR assay kit (Thermo Fisher Scientific).

Whole-genome sequencing was performed at the Genomics Unit from the Centre for Genomic Regulation (CRG) with a HiSeq2500 machine. Libraries were prepared using the NEBNext Ultra DNA Library Prep kit for Illumina (New England BioLabs, USA) according to the manufacturer’s instructions. All reagents subsequently mentioned are from the NEBNext Ultra DNA Library Prep kit for Illumina if not specified otherwise. One microgram of gDNA was fragmented by ultrasonic acoustic energy in Covaris to a size of ∼ 600 bp. After shearing, the ends of the DNA fragments were blunted with the End Prep Enzyme Mix, and then, NEBNext Adaptors for Illumina were ligated using the Blunt/TA Ligase Master Mix. The adaptor-ligated DNA was cleaned up using the MinElute PCR Purification kit (Qiagen, Germany), and a further size selection step was performed using an agarose gel. Size-selected DNA was then purified using the QIAgen Gel Extraction Kit with MinElute columns (Qiagen), and library amplification was performed by PCR with the NEBNext Q5 Hot Start 2× PCR Master Mix and index primers (12–15 cycles). A further purification step was done using AMPure XP Beads (Agentcourt, USA). Final libraries were analyzed using Agilent DNA 1000 chip (Agilent) to estimate the quantity and check size distribution, and they were then quantified by qPCR using the KAPA Library Quantification Kit (KapaBiosystems, USA) prior to amplification with Illumina’s cBot. Libraries were loaded and sequenced 2 × 125 on Illumina’s HiSeq2500. Base calling was performed using the Illumina pipeline software. In multiplexed libraries, we used 6-bp indexes. De-convolution was performed using the CASAVA software (Illumina, USA).

### Raw sequencing data analysis

Raw sequencing data was inspected with FastQC v0.11.5 (http://www.bioinformatics.babraham.ac.uk/projects/fastqc/). Paired-end reads were filtered for quality below 10 or 4 bp sliding-windows with average quality per base of 15 and for the presence of adapters with Trimmomatic v0.36 [62]. A minimum read size was set to 31 bp. To discard the possibility that higher quality thresholds would change our results, the trimming process was repeated for the five strains with lower coverage, using a minimum quality threshold of 28. After read mapping and variant calling (see the “Read mapping and variant calling” section), the main difference was the read depth of the called variants, which was lower in the stricter filter, decreasing the number of variants passing the filtration process (Additional file 12: Table S8). These results suggest that for this data, the quality threshold of 15 represents a good compromise between the read quality and the depth of coverage.

The K-mer Analysis Toolkit (KAT, [38]) was used to get the 27-mer frequency (default *k*-mer size) and GC content of each library. This program was also used to inspect the representation of each 27-mer in the respective reference genome. The genome assemblies used as reference were as follows: *C. albicans* assembly 22 haplotype A and B in separate [44], *C. orthopsilosis* 90-125 ASM31587v1 [63], *C. metapsilosis* chimeric genome assembly [2], *C. parapsilosis* ASM18276v2 [61], *C. tropicalis* ASM633v3 [64], and *C. dubliniensis* ASM2694v1 [65].

### Read mapping and variant calling

Read mapping of each sequencing library to the respective reference genome assembly was performed with BWA-MEM v0.7.15 [66]. It is important to note that for *C. albicans*, read mapping was performed in separate on haplotypes A and B. Picard integrated in GATK v4.0.2.1 [67] was used to sort the resulting file by coordinate, as well as to mark duplicates, create the index file, and obtain the mapping statistics. The mapping results were visually inspected with IGV version 2.4.14 [39]. Mapping coverage was determined with SAMtools v1.9 [68].

SAMtools v1.9 [68] and Picard integrated in GATK v4.0.2.1 [67] were used to index the reference and create its dictionary, respectively, for posterior variant calling. GATK v4.0.2.1 [67] was used to call variants with the tool HaplotypeCaller set with “--genotyping-mode DISCOVERY --standard-min-confidence-threshold-for-calling 30 -ploidy 2”. The tool VariantFiltration of the same program was used to filter the vcf files with the following parameters: “-G-filter-name “heterozygous” -G-filter “isHet == 1” --filter-name “BadDepthofQualityFilter” -filter “DP <= 20 || QD < 2.0 || MQ < 40.0 || FS > 60.0 || MQRankSum < -12.5 || ReadPosRankSum < -8.0” --cluster-size 5 --cluster-window-size 20.” In order to determine the number of SNPs/kb, a file containing only SNPs was generated with the SelectVariants tool. For this calculation, only positions in the reference with 20 or more reads were considered for the genome size, and these were determined with bedtools genomecov v2.25.0 [69].

### Heterozygous and homozygous blocks definition

To determine for each highly heterozygous strain the presence of heterozygous and LOH blocks, we adapted the procedure validated by Pryszcz et al. [2]. Briefly, bedtools merge v2.25.0 [69] with a window of 100 bp was used to define heterozygous regions, and by opposite, LOH blocks would be all non-heterozygous regions in the genome. The minimum LOH and heterozygous block size was established at 100 bp. All regions that did not pass the requirements to be considered LOH or heterozygous blocks were classified as “undefined regions.” No filter for coverage was applied due to the low coverage of some libraries. For hybrid strains, the current divergence between the parentals was calculated by dividing the number of heterozygous positions by the total size of heterozygous blocks.

We used a different method to define heterozygous blocks as compared to other studies because we consider that window-based approaches overestimate heterozygous block sizes, as window boundaries will rarely coincide with real heterozygous block boundaries. To confirm that this different approach (and not differences in variant calling) explains differences in levels of heterozygosity described in previous studies for the same strains, we repeated the analysis of SC5314 using the methodology described by Hirakawa et al. and Bensasson et al. (sliding-window approach), expecting to recover results similar to theirs [32, 36]. As shown in Additional file 13: Table S9, applying Hirakawa et al.’s method, we estimate ~ 80% heterozygosity for SC5314, which is similar to what they estimated [32]. In the case of Bensasson et al., although they do not indicate an estimation of heterozygosity, they report that the average of heterozygosity in windows with more than 0.1% SNPs is > 0.4%, and we estimated it to be 0.5%, which is consistent [36]. Furthermore, we could observe that depending on the window size, different estimations were made (Additional file 13: Table S9), with shorter windows estimating lower levels of heterozygosity, and apparently being more precise. This indicates that our results are not an artifact of variant calling.

### Comparison of SNPs across different strains

If *C. albicans* is a hybrid, we would expect that the majority of heterozygous SNPs in heterozygous blocks would be shared by the different strains, as this would mean that they were present before they diverged. On the other hand, in LOH blocks, we would expect exactly the opposite, as the majority of heterozygous SNPs should correspond to new acquired mutations. To check this scenario, we compared the heterozygous and LOH blocks of four strains from different clades. Based on the phylogeny described by [34], we decided to consider three groups of strains, which worked as replicates of the analysis (Fig. 2a). Each group was comprised of two strains from each side of the first node of divergence of *C. albicans* strains. To ensure that the results were not influenced by events of recombination between different clades, we only selected strains that did not present signs of admixture with other clades according to Ropars et al. [34]. The first group of strains comprised CEC4492 (clade 11) and SC5314 (clade 1) from one side, and CEC4497 (clade 4) and CEC5026 (clade 9) from the other side. The second one comprised CEC1426 (clade 11) and CEC3544 (clade 1) from one side, and CEC3716 (clade 4) and CEC3533 (clade 9) from the other side. Finally, the third group comprised CEC3601 (clade 11) and CEC3560 (clade 1) from one side, and CEC2021 (clade 2) and CEC3557 (clade 3) from the other side. For each group, we obtained the intersection of their LOH blocks and the intersection of their heterozygous blocks using bedtools intersect v2.25.0 [69]. For each intersection, we inspected the heterozygous positions in each strain and compared them with the observed genotypes in the other three clades. For each position, we reconstructed the most parsimonious scenario by assessing all possible mutational paths and choosing the one with the lower number of inferred mutations. When this scenario pointed to a similar heterozygous genotype in the common ancestor of the four strains, this position was assigned as “ancestral” for that strain. In case it would point to a different genotype, this heterozygous position was assigned as “recent.” When it was not possible to find a unique best scenario, the position was assigned as “ambiguous.” We also performed this analysis considering only blocks with an intersection > 100 bp. Furthermore, another analysis of both LOH and heterozygous block intersections was performed separately for coding and non-coding regions. For that, bedtools intersect v2.25.0 [69] was used to obtain for each intersection the blocks with at least one overlap with coding regions annotated for *C. albicans* assembly 22 and available at Candida Genome Database [70]. Detailed information on the size of the intersection and positions considered for analyses are detailed in Additional file 7: Table S5.

### Phylogenetic analysis considering the two haplotypes

The phylogenetic analysis of *C. albicans* considering the two haplotypes was performed individually for each of the mentioned groups of strains. Thus, for each group, we selected the intersection of heterozygous blocks (comprising the two haplotypes), which were defined as described above. To make sure that in the final alignment haplotypes A and B blocks were correctly concatenated, only regions overlapping SC5314 heterozygous blocks were taken into consideration, because they are the only ones correctly phased in the genome assembly. Then, for each strain, the respective homozygous SNPs were substituted in the reference genome. To separate the two haplotypes, HapCUT2 [71] was used to phase the heterozygous variants. For each block, the most similar haplotype to SC5314 haplotype A was considered A, while the most distant one was considered B. Positions with INDELs in at least one of the strains were excluded. In the end, for each group, we had an alignment of the two haplotypes of each strain. A maximum likelihood tree representative of each alignment was obtained with RAxML v8.2.8 [72], using the GTRCAT model and 1000 bootstraps. Midpoint rooting method was used to root the trees.

### Comparison of SNPs between *C. albicans* and *C. africana* strains

To assess whether *C. africana* was one of *C. albicans* parental lineages, we compared the heterozygous positions of *C. albicans*, with the homozygous positions of *C. africana.* As *C. albicans* genome was phased based on SC5314 [44], proved in this work to be a hybrid strain, only heterozygous regions in this strain are expected to represent the two parental haplotypes in the reconstructed phased genome. This means we can only trust that haplotype A of the reference genome corresponds always to the same parental in SC5314 heterozygous blocks. Therefore, for this analysis, we considered the intersection of these blocks with the heterozygous positions of each of the groups of *C. albicans* strains (check previous sections for details) and with the homozygous blocks defined for each *C. africana* strain. This analysis was performed independently for each group and each *C. africana* strain. Then, for each of these regions, we counted how many ancestral or recent positions identified in each of the four *C. albicans* strains of a given group (check previous sections for details) were shared (the same genotype was found in *C. albicans* and *C. africana*), or corresponded to a haplotype (haplotype A or B was present in both strains), or were undefined (none of the previous options was observed). Details on this analysis can be found in Additional file 11: Table S7.

## Supplementary information

**Additional file 1: Figure S1.** 27-mer frequency plots for SC5314, CEC4492, CEC4497, CEC5026 (*C. albicans*), MCO456, s425, s433, s498 (*C. orthopsilosis* hybrids), CP367 (*C. metapsilosis* hybrid), CBS6318, GA1, BC014 (*C. parapsilosis* non-hybrids), s428 (*C. orthopsilosis,* non-hybrid parental lineage A), 60–13 (*C. parapsilosis* non-hybrid), ATCC200956 and CSPO (*C. tropicalis* non-hybrid), and their respective presence (red) or absence (black) in the respective reference genome (plots were obtained with KAT [38]).

**Additional file 2: Table S1.** Detailed information on read coverage, mapping statistics and genomic variability of all *C. albicans* strains when aligned to the haplotype A*.*

**Additional file 3: Table S2.** Detailed information on genomic variability of all analyzed non-*albicans* strains when aligned to the respective genome assemblies.

**Additional file 4: Figure S2.** Coverage tracks for illustrative genomic regions of **A)***C. albicans* strains; **B)***C. albicans* strains with LOH towards different parents highlighted in the red box; **C)***C. orthopsilosis* hybrid strains; and **D)***C. metapsilosis* hybrid strain. Colors indicate polymorphic positions. Positions with more than one color correspond to heterozygous variants. Visualizations were performed with IGV [66].

**Additional file 5: Table S3.** Detailed information on the variability observed in heterozygous and LOH blocks for all *C. albicans* strains when mapped to haplotype A.

**Additional file 6: Table S4.** Union and intersection of heterozygous blocks in each chromosome.

**Additional file 7: Table S5.** Detailed results of the inference of heterozygous SNPs ancestrality for the three groups of *C. albicans* strains.

**Additional file 8: Figure S3.** Distribution of ancestral SNPs/kb in group 1 (left), group 2 (center), and group 3 (right).

**Additional file 9: Figure S4.** Maximum likelihood phylogeny of the aligned reconstructed haplotypes A and B for the intersection of heterozygous blocks > 100 bp of **A)** group 2; and **B)** group3.

**Additional file 10: Table S6.** Detailed information on read coverage, mapping statistics and genomic variability of all *C. africana* strains when aligned to the haplotype A of *C. albicans.*

**Additional file 11: Table S7.** Detailed results of the inference of heterozygous SNPs ancestrality in comparison to *C. africana* for the three groups of *C. albicans* strains.

**Additional file 12: Table S8.** Comparison of the variant calling results obtained with trimming quality thresholds of 15 and 28.

**Additional file 13: Table S9.** Level of heterozygosity of SC5314 considering a sliding-window approach.

## Data Availability

All data generated or analyzed during this study are included in this published article, its supplementary information files, and publicly available repositories. Specifically, the datasets generated during the current study are available in NCBI under the BioProject PRJNA555042 [73]. Data made publicly available by other projects are available in NCBI under the BioProject numbers PRJNA432884, PRJEB27862, PRJEB4430, PRJNA322245, PRJEB1698, PRJEB1685, PRJNA326748, PRJNA194439, PRJEA83665, PRJEA32889, PRJNA13675, and PRJEA34697 [74–85]. *C. albicans* assembly 22 is available in the Candida Genome Database [86].
