# Supplementary figures and images for: Genomic evidence for a hybrid origin of the yeast opportunistic pathogen Candida albicans
Source: BMC Biol. 2020 May 6;18:48. doi: 10.1186/s12915-020-00776-6 (PMC7204223; doi:10.1186/s12915-020-00776-6)

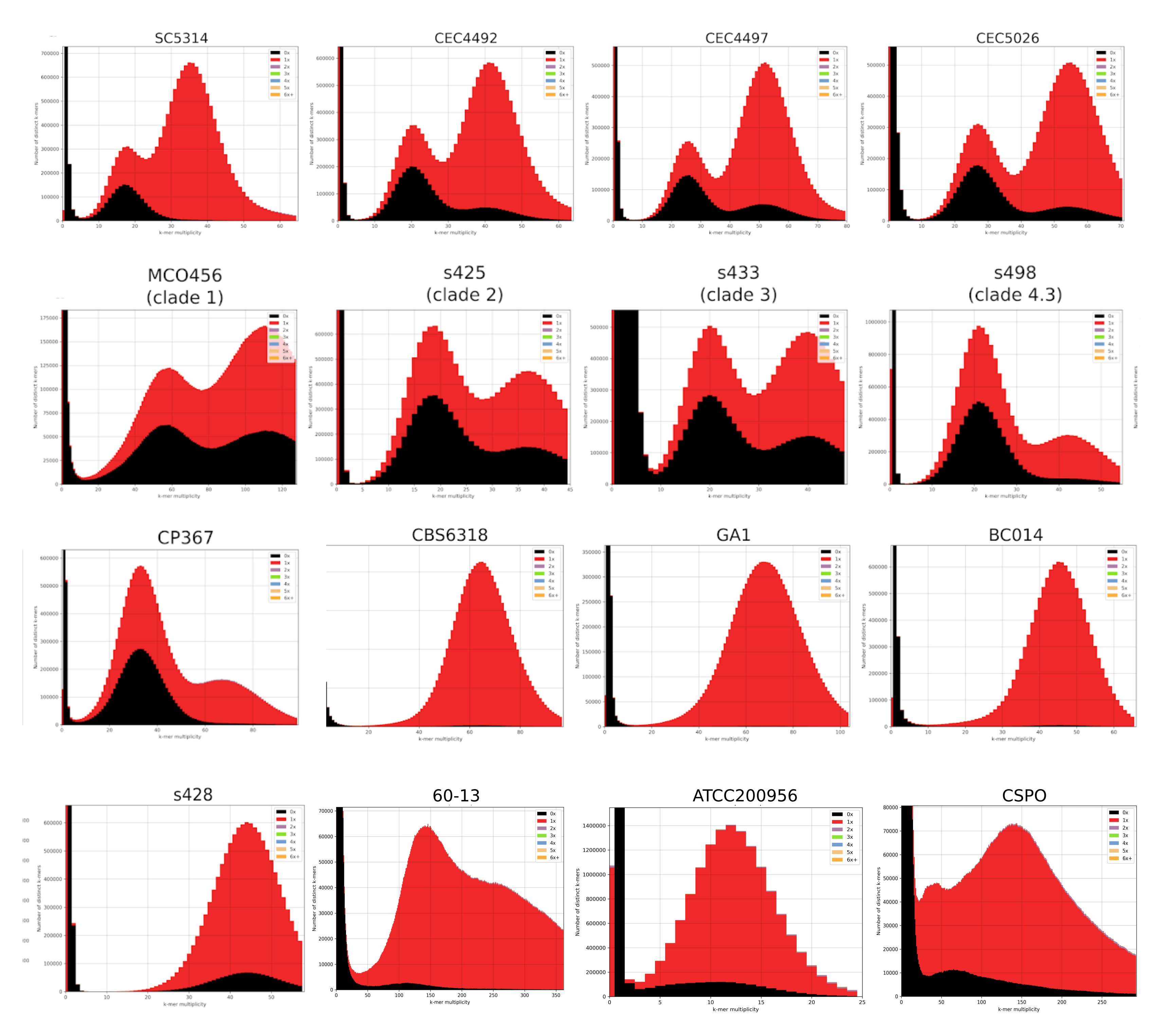

Supplement: Supplementary file 1 — Additional file 1: Figure S1. 27-mer frequency plots for SC5314, CEC4492, CEC4497, CEC5026 (C. albicans), MCO456, s425, s433, s498 (C. orthopsilosis hybrids), CP367 (C. metapsilosis hybrid), CBS6318, GA1, BC014 (C. parapsilosis non-hybrids), s428 (C. orthopsilosis, non-hybrid parental lineage A), 60–13 (C. parapsilosis non-hybrid), ATCC200956 and CSPO (C. tropicalis non-hybrid), and their respective presence (red) or absence (black) in the respective reference genome (plots were obtained with KAT [38]). [file 12915_2020_776_MOESM1_ESM.png]

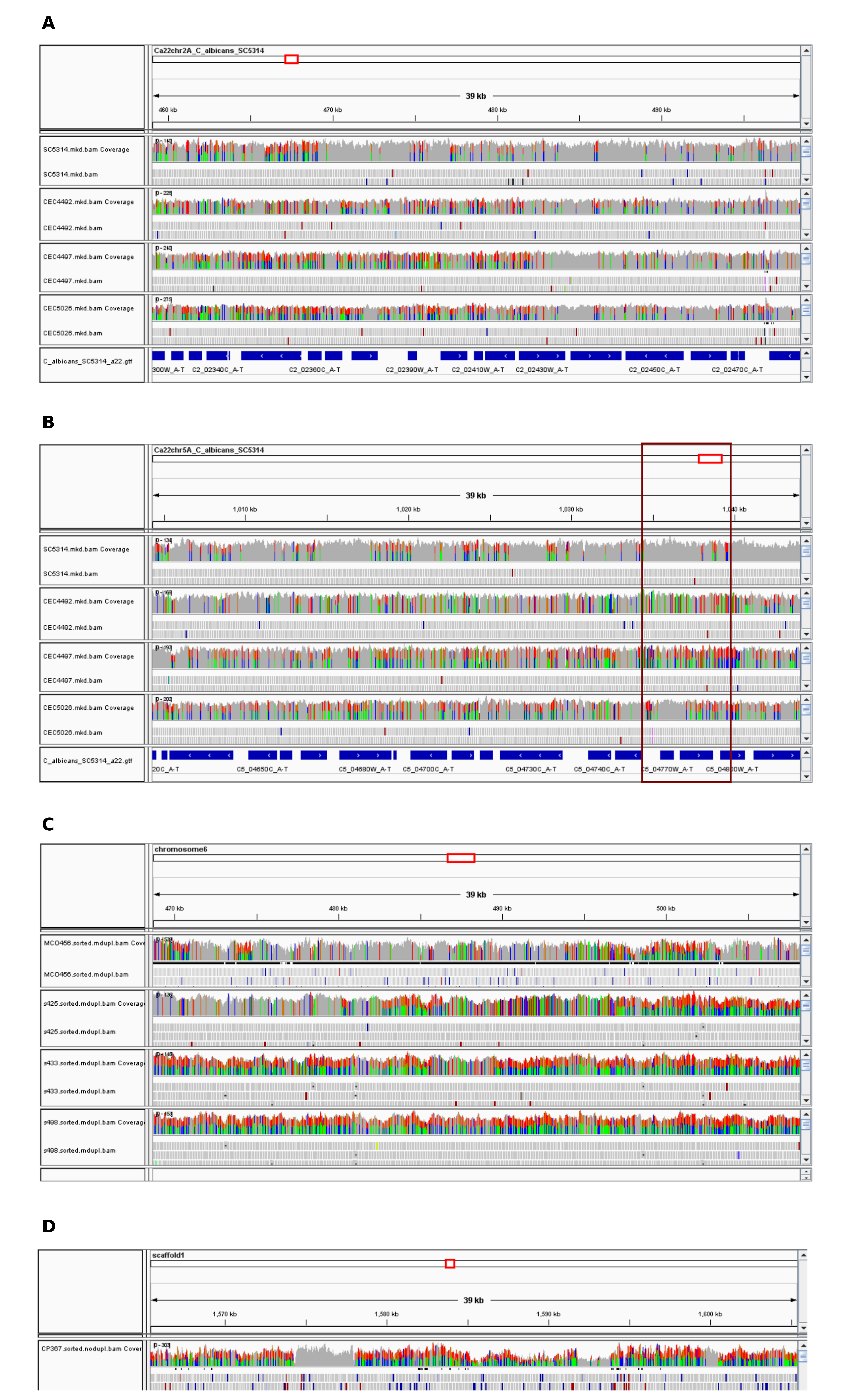

Supplement: Supplementary file 4 — Additional file 4: Figure S2. Coverage tracks for illustrative genomic regions of A)C. albicans strains; B)C. albicans strains with LOH towards different parents highlighted in the red box; C)C. orthopsilosis hybrid strains; and D)C. metapsilosis hybrid strain. Colors indicate polymorphic positions. Positions with more than one color correspond to heterozygous variants. Visualizations were performed with IGV [66]. [file 12915_2020_776_MOESM4_ESM.png]

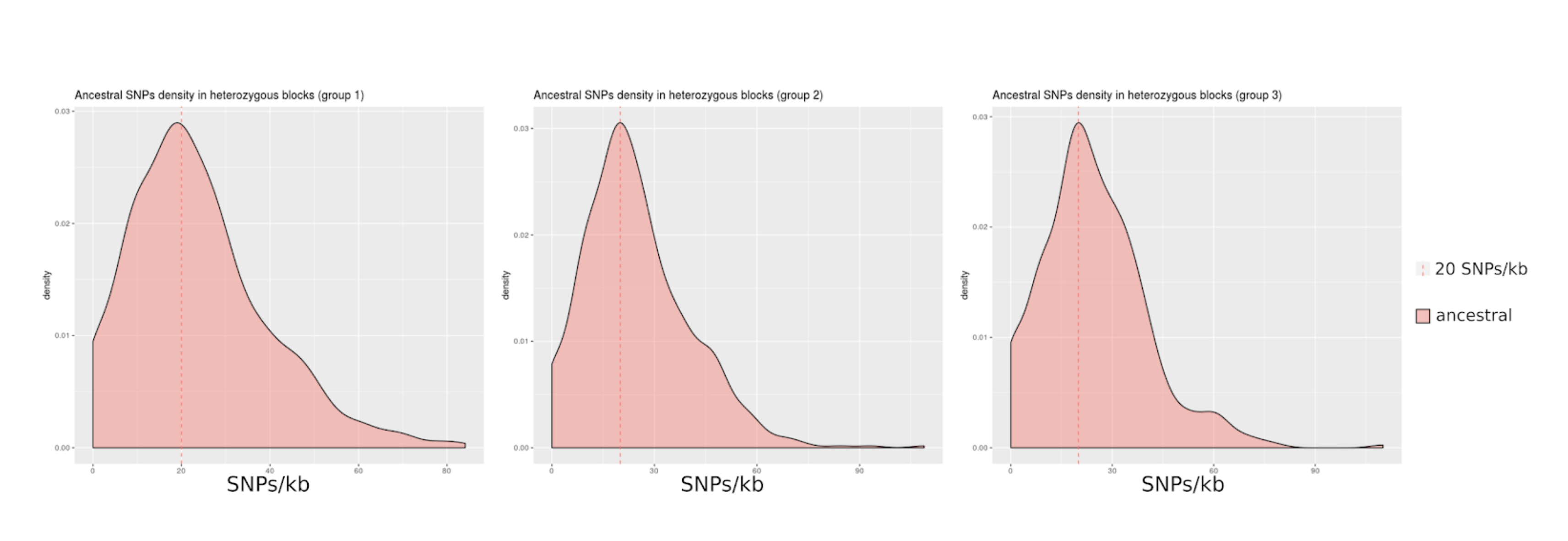

Supplement: Supplementary file 8 — Additional file 8: Figure S3. Distribution of ancestral SNPs/kb in group 1 (left), group 2 (center), and group 3 (right). [file 12915_2020_776_MOESM8_ESM.png]

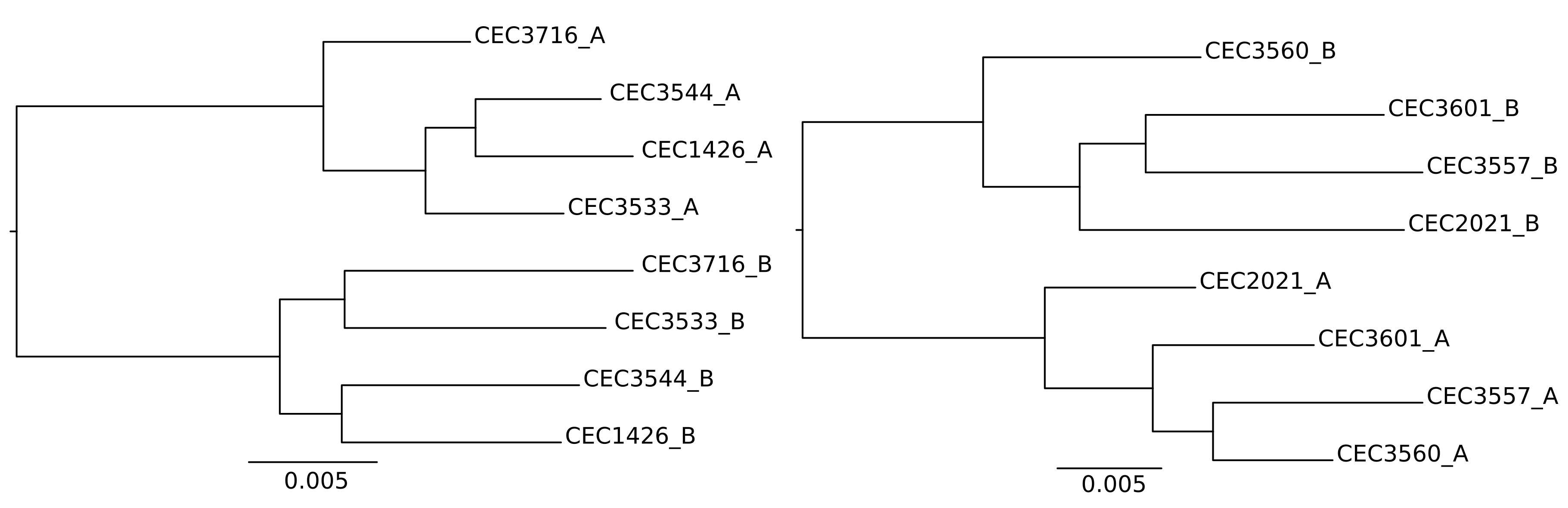

Supplement: Supplementary file 9 — Additional file 9: Figure S4. Maximum likelihood phylogeny of the aligned reconstructed haplotypes A and B for the intersection of heterozygous blocks > 100 bp of A) group 2; and B) group3. [file 12915_2020_776_MOESM9_ESM.png]
